# Challenging the Neo-Anthropocentric Relational Approach to Robot Rights

**DOI:** 10.3389/frobt.2021.744426

**Published:** 2021-09-14

**Authors:** Henrik Skaug Sætra

**Affiliations:** Faculty of Computer Sciences, Engineering and Economics, Østfold University College, Halden, Norway

**Keywords:** anthropocentrism, ethics, moral standing, robots, rights, social robots, robot rights, neo-anthropocentrism

## Abstract

When will it make sense to consider robots candidates for moral standing? Major disagreements exist between those who find that question important and those who do not, and also between those united in their willingness to pursue the question. I narrow in on the approach to robot rights called relationalism, and ask: if we provide robots moral standing based on how humans relate to them, are we moving past human chauvinism, or are we merely putting a new dress on it? The background for the article is the clash between those who argue that robot rights are possible and those who see a fight for robot rights as ludicrous, unthinkable, or just outright harmful and disruptive for humans. The latter group are by some branded human chauvinists and anthropocentric, and they are criticized and portrayed as backward, unjust, and ignorant of history. Relationalism, in contrast, purportedly opens the door for considering robot rights and moving past anthropocentrism. However, I argue that relationalism is, quite to the contrary, a form of neo-anthropocentrism that recenters human beings and their unique ontological properties, perceptions, and values. I do so by raising three objections: 1) relationalism centers human values and perspectives, 2) it is indirectly a type of properties-based approach, and 3) edge cases reveal potentially absurd implications in practice.

## Introduction

If we provide robots moral standing because of how humans relate to them, are we moving past human chauvinism, or are we merely putting a new dress on it? Questions related to moral standing go back a long way (Sætra, 2019), and they always trigger strong emotions and require that we deal with both difficult and fundamental questions. Different types of humans–demarcated by color, sex, and a range of other arbitrary attributes of questionable moral relevance–have fought tough battles for being recognized as of equal, or at least some, value. Other entities, such as animals, cannot fight for their own rights, but humans have still taken it upon themselves to fight for their rights (Regan, 2004). Even rivers, trees, and the abiotic parts of the environment have been the subject of a fight for rights because humans have decided to become their champions (Stone, 1972).

The latest installment in the saga of rights–the fight for other’s rights–are robots. While robots are somewhat new, the debates they give rise to are arguably not, as they draw upon and continue debates from environmental ethics. While not new, the question of how artificial entities fit into these old debates is attracting increased attention (Harris and Anthis, 2021). Old arguments, on old battlegrounds, are rehashed, as robot champions (champions for the rights of robots) clash with those who call the fight for robots right ludicrous, unthinkable, or just outright harmful and disruptive of the fight for equal get for all humans (Birhane and Van Dijk, 2020a; 2020b). The latter group is by some branded human chauvinists since their arguments are considered to be anthropocentric, and they are consequently criticized and labeled as both backward, unjust, and ignorant of history.

One particular form of argument for imagining robot rights is relationalism, with Coeckelbergh. (2010), Coeckelbergh. (2011), Jones. (2013), Gunkel. (2018b), and Gellers. (2020) as some of its champions in arms. Thinking otherwise, Gunkel calls it, when he argues that relational ethics opens the door for seriously considering robot rights and taking a step or two past anthropocentrism. In this article, I challenge the implicit and at times explicit claim that relationalism allows us to move past anthropocentrism, as I argue that the approach is in fact a form of neo-anthropocentrism that recenters human beings and their unique ontological properties, perceptions, and values and that this is quite the opposite of the stated purpose of this purported thinking outside the box (Gunkel, 2018b). I do so by raising three objections: 1) relationalism centers human values and perspectives, 2) it is indirectly a type of properties-based approach, and 3) edge cases reveal potentially absurd implications in practice.

My goal is thus to challenge the proponents of this approach to clarify and further develop their theories, and others have similarly claimed that relationism “leaves us with many unresolved questions” (Tavani, 2018). I will, however, not pursue the question of whether or not the relational approach is more useful or better than the alternatives, as the purpose is to highlight issues related to the anthropocentric nature of the approach. This also means that I will not be evaluating the different varieties and the nuances of the various philosophical foundations used by the different researchers in this tradition, beyond what is required for establishing whether or not the emerging tradition–as a tradition–is anthropocentric.

In order to evaluate the nature of relationalism, in *Anthropocentrism and the Others* I examine the nature of anthropocentrism and non-anthropocentrism. More importantly, I highlight the importance of examining the different types of each, because the umbrella terms “anthropocentrism” and “non-anthropocentrism” themselves contain too much variation to be philosophically meaningful. In *The relational Turn as Neo-Anthropocentrism* I move on to relationalism, to briefly present how its proponents present the approach before I proceed to examine it in light of the types discussed in *Anthropocentrism and the Others*. I end this section by presenting the three objections which together constitute my challenges to relationalism.

## Anthropocentrism and the Others

As the starting point for the examination of the nature of the relational turn in the robot rights discourse, the field of environmental ethics provides a range of applicable tools and concepts. It could even be argued that the question of the moral standing of robots is a part of environmental ethics, and does not necessitate new forms of ethics such as robot ethics. Environmental ethics is, after all, at times understood as the examination of how moral thinking and action can be expanded both beyond humans and beyond the present (Nolt, 2014). Just as the robot rights movement is often perceived as a form of unwarranted and misdirected activism (Birhane and Van Dijk, 2020a; Birhane and Van Dijk, 2020b), the same often goes for environmental ethicists, at times labeled “treehuggers,” anti-humanists or misanthropes who fight for the rights of animals and the natural world at the expense of human beings (Drengson, 1995; Kopnina et al., 2018; Rottman et al., 2021). Such a denouncement is, however, based on the erroneous notion that there is a “hierarchy of ethics” and that all research should be directed to whichever problems the critics consider to be more important than considering robot–or environmental–rights (Sætra and Fosch-Villaronga, 2021).

Key concepts related to the moral standing of robots are moral community, moral agency, and moral patiency (Nolt, 2014). All entities that are deemed worthy of moral consideration belong to the moral community, and anyone who has a claim on moral consideration is a moral patient. Some entities will have such a claim and an associated moral duty, and these are considered moral agents. The moral community is here considered a purely hypothetical construct, and any type of moral community is theoretically possible. One theory might argue that only humans have a claim for moral consideration, while another might argue that humans are not even parts of the moral community.

Robots can, in theory, certainly be considered parts of our moral community, but few as of yet have argued that they are full-fledged moral agents. Most existing theories will consider humans moral agents while including some other entities as moral patients with various claims to moral consideration. It is important to stress that no universally accepted definitions of which traits warrant moral patiency exist (Gellers, 2020; Gunkel, 2018b), and this is one of the very reasons for the emergence of the relational approach, as we will later see. Neither are the criteria for moral agency sufficiently clear to serve as the basis of agreement between moral philosophers of various stripes. However, I’ll argue that it is reasonable to posit that if we are to provide an entity with a duty to consider the moral claims of others, they must have a least the semblance of the sensory and cognitive capacities to do so–they must have moral competence (Nolt, 2014). As argued by Næss. (1989) humans are the first species with the capacity to understand how their behavior affects other beings and consequently change this behavior to achieve some form of equilibrium. Humans can, he argues, “perceive and care for the diversity of their surroundings” (Næss, 1989, p. 23), even if they arguably do not always do so.

This framework allows us to examine how different people ascribe moral standing to different forms of entities, potentially including robots. Rather than focusing on the resulting ascription of moral standing to various entities, I’m concerned with how the theorists most clearly associated with the “relational turn” in robot ethics arrive at the possibility of rights for robots, and in particular whether or their approach is less anthropocentric than other approaches. In order to achieve this, a somewhat roundabout trip into the murky definitional waters surrounding anthropocentrism and non-anthropocentrism is required. That is because, as we shall see, these terms are often used in a confusing and non-specific manner.

### Anthropocentrism

Ethical theories that assume the centrality of humans in any consideration of moral standing or value are often referred to as anthropocentric (Nolt, 2014). Posthumanism–focusing on the “decentering of humans”–and the biospherical egalitarianism of Næss, for example, might both be argued to entail clear rejections of anthropocentrism (Næss, 1973; Meyer, 2001; Braidotti, 2013). Robot rights is another phenomenon that might at first seem to be–and is often argued to be–non-anthropocentric. Concluding that they are non-anthropocentric is, however, premature, as I will argue that even theories that do not consider humans to be most–or the only things–valuable can clearly be anthropocentric, depending on their method of ascribing moral standing to others. To make this point, the most important types of anthropocentrism must be examined.^1^ I rely on Nolt (2014) terminology for distinguishing between axiological and ethical anthropocentrism in what follows.

#### Axiological Anthropocentrism

While there may be many reasons for humans putting themselves at the center of any moral examination, Nolt. (2014) points to the emergence of monotheism as the key factor which led to a focus on humanity as a meaningful group, rather than mere humans as contained in distinct cultures and smaller groups. He then connects anthropocentrism and monotheism to the notion of human rights, which is arguably one of the most important manifestations of anthropocentric ethics prevalent in modern societies (Zimmerman, 1985). Much like Aristotle had done before them, late antiquity Western philosophers, working in the age of emerging monotheism, saw the world as “an exquisitely designed hierarchical structure in which all things had God-given values and purposes” (Nolt, 2014, p. 64). All things, according to this line of thinking, exist to serve the needs of “higher” entities. Water serves the needs of plants, which serve the need of animals, which serve the need of humans, for example. A central example of this line of thinking is the idea of a great chain of being (Lovejoy, 1936). In the original chain angels and God superseded humans, while the modern secular and anthropocentric version could be argued to place humans at the pinnacle.

This kind of anthropocentrism, with humans at the center and everything else assigned value by how it serves human needs, is referred to as axiological anthropocentrism (Nolt, 2014). If humans, for example, tend to see something of themselves–something that they value–in robots, this could become the basis of offering such entities some form of moral consideration, or protection (Darling, 2016a). This also relates to the notion that how we treat other entities impacts us. If, for example, humans somehow hurt themselves by mistreating animals, or robots (Darling, 2016a), this could give rise to both moral and legal protection of such entities. Not for the entitie’s sake, but for ours. The moral standing and value of entities are, according to this theory, assigned by and for human purposes. For us, by us. While somewhat similar to the religious view that things have value according to God’s desires and purposes, it is clearly distinct, as no God or gods serve any necessary purpose in axiological anthropocentrism. If humans find a purpose for God, however, God will be valued accordingly, but not the other way around.

#### Ethical Anthropocentrism

A different form of anthropocentrism, one that is often conflated with the axiological variety, is ethical anthropocentrism. This is a theory that encompasses the view that humans are morally considerable, and most other things are not (Nolt, 2014). The strict variety states that only humans are morally considerable, while other varieties assign some–but not much–value to some non-human entities. It is easy to see why axiological and ethical anthropocentrism are often conflated, but it is still important to distinguish between them. Axiological anthropocentrism allows for a far more inclusive moral community than ethical anthropocentrism, provided that humans find value in other beings, or that humans simply find value in providing other beings with moral standing. The common denominator is that humans are centered, as they are either of superior moral standing or the only cause of moral standing provided to others. The instrumental approach to robot rights is often based on ethical anthropocentrism, and, for example, the notion that robots should be slaves (Bryson, 2010), is based on the idea that they do not have moral worth, even if they can indeed be useful. According to such positions, they could be said to have instrumental value to humans, but no intrinsic value. Ethical anthropocentrism is also clearly linked to the idea of human chauvinism, which is, according to some, deeply embedded in western culture and consciousness (Seed, 1988).

### Non-Anthropocentrism

While we are currently seeing a growing concern for the environment–in the shape of animals, the climate, or various ecosystems–it is still considered relatively radical to argue in favor of non-anthropocentric ethical theories. After all, a whole lot of those concerned with the climate and biological diversity, for example, make few efforts to hide the fact that their concerns stem from the negative effects for human beings if the environment is impoverished or changed in ways unfavorable to human flourishing. Truly non-anthropocentric theories must argue in favor of the worth of nature regardless of how nature impacts humans, and as with anthropocentrism, there are several types of non-anthropocentrism.

#### Rights and Ontology-Based Ethical Non-Anthropocentrism

One apparent way to move past anthropocentrism is to provide other entities with rights. I will only consider the approach that assumes that the entities that receive rights are capable of being bearers of rights, and not an entirely legalistic approach that ascribes “rights” to nature, corporations, etc., for merely instrumental purposes. Robots can, Sætra (2021a) argues, certainly be considered as some sort of limited liability corporations if this serves socio-political needs, but this must not be conflated with the notion that legal status also provides moral status.

Peter Singer and Tom Reagan and Singer. (1976) are two well-known proponents of animal rights, but others have also asked whether, for example, trees, rivers, or entire ecosystems have rights (Stone, 1972). The question given rise to by all these approaches is, however: what are rights derived from? Answers range from philosophy, deities, natural law, the use of (human) reason, etc. One particular approach, which is the loci of this article, is one where rights are ascribed on the basis of the nature of relationships, and the examination of this approach will be saved for the next chapter.

One kind of non-anthropocentrism uses traits, or ontological features of entities, to argue that humans are not really special, and thus do not deserve a special moral status. These theories are often related to the problems of demarcation that arise as soon as someone attempts to defend ethical anthropocentrism by describing why humans are different from animals (Nolt, 2014). In a discussion of what distinguishes humans from machines, for example, Sætra (2019) examines a range of different properties, or traits, that have historically been used to distinguish humans from other entities. Reason, a soul, life, etc.–all these concepts fail, he argues, as the basis for clearly demarcating humans from others. When traits are tested as criteria for human value, marginal cases are often used to demonstrate the problems associated with the various criteria (Dombrowski, 1997; Nolt, 2014). For example, if reason is our criteria, how do we deal with the fact that some animals have more of it than some humans (Sætra, 2019)? Such a traits-based approach could arguably be both anthropocentric and non-anthropocentric, and also anthropocentric in two different ways. If traits are chosen in order to limit moral consideration to *Homo sapiens,* we get ethical anthropocentrism, while other approaches focus on traits shared by other entities as well–such as sentience–in which we might have axiological anthropocentrism.

Another way to potentially derive rights, obligations, and moral standing from a situation in which none exist is the contractualist approach (Hobbes, 1946). With this approach, rights are derived from consent based on contract, but neither the contract nor the consent need be explicit. In Hobbe’s social contract theory, for example, the contract can be considered a hypothetical thought experiment aimed at generating agreement of what reasonable people would agree to, and thus the contract is not taken to be an actual contract that each and every individual has actually agreed to (Sætra, 2014). While Sætra. (2014) argues that contractualism might lead to a type of environmentalism based on human self-interest, the social contract theorist Carruthers. (1992) has warned that if we extend our moral communities to encompass other entities, morality might be diluted. Despite such warnings, contractualism can potentially lead to rights for others, just as we have extended rights to animals and a range of other beings that cannot themselves be an active contracting party. One approach to a contractual approach to non-human rights is to have humans serve as curators or guardians (Sætra, 2014).

The notion of rights is a topic worthy of its own article, and as I am mainly concerned with understanding how the relational approach to moral standing relates to anthropomorphism, I save the rest of this discussion for *The relational Turn as Neo-Anthropocentrism*, in which the relational turn is examined in more detail. Before that, two non-anthropocentric varieties will provide more insight into just how we might justify the decentering of humans.

#### Axiological Non-Anthropocentrism

Axiological anthropocentrism ascribes value to entities according to how valuable they are perceived to be by humans. Humans are a relatively diverse bunch, however, and it is here important to be wary of the danger of conflating western values with human values (Gellers, 2020). Moving beyond the discussion of whose human values matter, the axiologically non-anthropocentric approach starts with the assumption that human values are not the only values (Nolt, 2014). Other entities might indeed have values as well, or things might at the very least potentially be good or bad for them. The problem with this theory, as compared to the anthropocentric variety, is that it is difficult to determine what the values of non-human entities really are. Three main approaches to discovering these are the hedonistic, preference-satisfaction, and objective welfare, all with their distinct strengths and obvious weaknesses (Nolt, 2014).

The hedonistic approach entails an emphasis on aggregate pleasure and pain, and it is often associated with the consequentialist variety of ethics referred to as utilitarianism. However, concerning moral standing, the ability to experience pleasure and pain is what matters, and sentientism is perhaps the most prominent variety in this category. The key objection to sentientism in the context of robot rights is that it once again entails examining the ontological status of subjects–here the capability of sentience. Furthermore, since it is most often biocentric (Nolt, 2014), it tends to exclude machines. However, critics argue that it is difficult to distinguish human pain and pleasure from what is “experienced” by a sophisticated machine, just as reason and other objective qualifiers also bring us into murky waters. This objection to biocentrism becomes increasingly relevant with modern advances in biomimetic robots (Winfield, 2012), and various robots built to model human emotions, homeostasis etc. (Cominelli et al., 2018; Man and Damasio, 2019).

Preference-satisfaction is a broader form of consequentialism in which entities may be thought to have interests beyond pleasure and pain, and what subjects themselves consider good and desire is what matters. But how do we uncover the preferences of entities that cannot speak or express themselves? Those who are capable of acting are helpful in that we might propose using the theory of revealed preferences from economic theory (Samuelson, 1948), but what about abiotic nature? And what about robots, who can both speak and act? This is where the question of agency comes up, and in this article, I adhere to the position that robots cannot as of now be said to be capable of owning and being responsible for their own actions, and consequently, I assume that their words and actions do not represent the robot’s own preferences in a meaningful way (Sætra, 2021a).

The final approach consists of basing one’s evaluation on some notion of objective welfare–an approach that might result from wanting to deduce what is good for, and thus assumed preferred, by entities. Næss (1989), for example, uses the notion of flourishing as a fundamental good for all entities. Highly useful for dealing with entities that neither speak nor act, but also for humans who might not realize their own best interests. Or at least so the paternalists might say.

#### Ethical Biocentrism

A different approach is one in which entities are quite simply regarded as origins of value. Inherent, or intrinsic, value is the term often used for this approach (Næss, 1989; Nolt, 2014), as their value is assumed to be entirely disconnected from their instrumental value to humans or to how humans imagine value. One example of such a theory is Arne Næss’s deep ecology, based on the notion of biocentric egalitarianism and an outright rejection of anthropocentrism and the superior moral value of humans (Næss, 1989).

As compared to the previous type of non-anthropocentrism, ethical biocentrism does not require us to uncover, or conjure up, the interests, preferences, etc., of other entities. Instead, they are considered valuable just because of being what they are, which is why the terms intrinsic or inherent value are often used. Midges and ticks, for example, have very little going for them in terms of obvious instrumental value for humans–particularly if people’s opinions about them, rather than the ecosystem services they provide–are the basis of ascribing value. However, in theories such as Næss’s deep ecology, even such beings are considered valuable by virtue of simply being what they are, and they are provided with the same rights to flourish like the rest of us. The details of what constitutes inherent or intrinsic value, and the differences used in describing who and what has such value, is beyond the scope of this article, and it suffices for now state that such approaches are effective in ascribing rights to animals, and at other parts of nature, while it has not been particularly useful for imagining robot rights.

## The Relational Turn as Neo-Anthropocentrism

The time has come to consider how to categorize theories belonging to what is often referred to as the “relational turn”^2^ in robot ethics (Gerdes, 2016). The goal of what follows is not to examine whether or not “relationalism” (Coeckelbergh, 2010), “social-relational ethics” (Coeckelbergh, 2010; Gunkel, 2018b; Harris and Anthis, 2021), or “ecological relationalism” (Jones, 2013) is right, wrong, beneficial, etc. Neither is it a deep philosophical examination of the nuances and differences between the various manifestations of relationalism beyond what is required to establish the fundamental approach shared by these theorists.

Rather, the goal is to examine whether relationalism is anthropocentric or non-anthropocentric, and using the terms established also determines more specifically which type most accurately describes it. The reason why uncovering this is of interest is that it is relevant to the discussions that emerge as soon as differences of opinion with regard to the possibility or desirability of robot rights surface. In such discussions, opponents of robot rights might argue that pursuing such questions is pointless, or even outright harmful, as more important questions related to human flourishing should be prioritized (Birhane and van Dijk, 2020b). Some arguing in favor of robot rights might then accuse the opponent of being human chauvinists, and either explicitly or implicitly indicate that the opponents are anthropocentric human chauvinists, while those open to robot rights are not. Whether or not these proponents are right is what I address in the following.

### The Relational Turn

What is here described as the relational turn refers to the idea that moral consideration should be premised on social relations rather than ontological or socio-political frameworks (Coeckelbergh, 2010). What I refer to as relationism is not the particular philosophy of one person, however, and I will, in general, refer to relationism as a tradition manifested through the work of Mark Coeckelbergh. (2010) Raya Jones. (2013), David Gunkel. (2018b), and Josh Gellers. (2020).

Relationalism is, however, not a new phenomenon, and it is often traced back to the relational approach of Arne Næss (Brennan and Lo, 2021; Næss, 1989). It is also closely related to care ethics (Donovan and Adams, 1996, Donovan and Adams, 2007), which emphasizes relationships, and both anthropocentric and non-anthropocentric varieties of care ethics have been proposed. Common for the traditional care ethics is that they are routinely criticized for their inability to extend rights to strangers–both humans and other types of others (Nolt, 2014).

One response to this is the relational ethic of Palmer. (2010), which is based on ecofeminism and its emphasis on relationships rather than individuals in isolation (Palmer, 2003). She, like the relationists in the robot ethics camp, suggests that responsibilities and moral standing are not just matters of capabilities, but also of our interactions with others. However, relationships are used differently by Palmer than by the robot ethicists, as she argues that actual interactions create responsibilities, more so than relatively abstract notions about how humans, in general, might be capable of forming relations with other entities. Consequently, I focus on relationalism as it is detailed in the robot ethics discourse, as robot relationalism and the traditional varieties just discussed are somewhat different.

The problem with traditional theories, Coeckelbergh. (2010) argues, is that they all–deontological, utilitarian, and partly virtue ethics–rely on what he calls “ontological features” of the entities in question. These are, for example, requirements related to biological life, rationality, sentience, etc., as we have already seen. He proceeds to argue that we need a “social ecology,” which is–much like Arne Næss deep ecology–based on the science of ecology combined with “Eastern worldviews.” This is also discussed at some length by Næss himself, and Jones. (2013), Jones. (2015), Gunkel. (2018b), Gellers. (2020), all emphasize the need for and potential utility of moving beyond traditional Western world-views in order to arrive at an improved understanding of how to understand moral standing and the nature of various others.

What becomes important, then, is the network of interactions and relations between entities, and not the entities in isolation and their properties.

The alternative approach I propose attempts to avoid the skepticism by replacing the requirement that we have certain knowledge about real ontological features of the entity by the requirement that we experience the features of the entity as they appear to us in the context of the concrete human-robot relations and the wider social structures in which that relation is embedded (Coeckelbergh, 2010, p. 14).

Rather than ascribing moral standing on the basis of characteristics of the entity–the properties-based approach–the very fact that we relate with other entities becomes the basis for obligations and claim for moral consideration (Gellers, 2020). As with the traditional types of relational theories, what matters is not necessarily whether or not the others are like us (Darling, 2016a), or if we see ourselves reflected in them (Sætra, 2021b), but rather how these others become actors in our social structures with which we interact. What I refer to as relationalism is, as mentioned, often referred to as social-relational ethics for this reason. This is also related to Arne Næss’s notion that self-realization entails coming to see ourselves as nodes in a relational total-field image (Næss, 1989). This leads to identifying with the other nodes in the field, and this is in theory accompanied by an acknowledgment of how all is, in reality, one, and all of value. While deep ecology opens for including both biotic and abiotic nature in this field, artificial life has no obvious function in this network. In robot relationalism, the fact that we relate to robots is taken as an indication that these entities are, in fact, nodes of value due to these relations.

The key argument, as presented by Gunkel. (2018b), is that our evaluation of what another entity is, in a moral context, depends on how it is treated and not some isolated consideration of what the thing in isolation is. In particular, it is important that the other is not simply reduced to a reflection of ourselves and some sort of alter-ego which is perceived as valuable because of its likeness to us (Gunkel, 2018a). Gunkel draws heavily on Levinasian philosophy in his explorations of the potential for robot rights, and it is interesting to note how he argues that it is important to “break free from the gravitational pull of Levinas’s own anthropocentric interpretations” (Gunkel, 2018a, p. 97). In his reinterpretation of Levina’s philosophy in a way that opens for considering robots to be meaningful others–as Levinas himself does not do–the question is whether Gunkel simply develops a new kind of anthropocentric theory or if he arrives at a non-anthropocentric theory. The objections which are shortly presented suggest that what has occurred is a move from ethical to axiological anthropocentrism and not a move to non-anthropocentrism.

Gellers. (2020) explores both the legal and moral status of robots, and while the former is outside the scope of this article, his considerations regarding the latter are largely in line with the preceding authors. He explicitly argues that the relational ethic proposed by Levinasian scholars (and he here includes Coeckelbergh and Gunkel) is both promising and that it has moved old debates, but also that it might go too far in abandoning the role of properties. This–the role of properties in our encounters with others–is also the very basis of one of my challenges.

What is of most interest here is not the nuances of the different varieties of relationalism, but whether or not relationalism really succeeds in moving us past anthropocentrism, or if it is instead a new type of anthropocentrism.

### Neo-Anthropocentric Relationalism

On the basis of the preceding considerations, I now turn to an explanation of why I argue that relationalism is a neo-anthropocentric ethical theory. Rather than providing the means to move beyond anthropocentrism and the traits-based approach, I argue, based on the objections presented below, that the theory is both anthropocentric and dependent on traits-based considerations. In addition, the theory is faced with a practical challenge related to its potential use in practice. In the following I outline three main challenges for relationalism: 1) relationalism centers human values and perspectives, 2) it is indirectly a type of properties-based approach, and 3) edge cases reveal potentially absurd implications in practice.

#### Human-Centered Relations

My first objection is that relationalism is arguably deeply anthropocentric because moral standing is derived exclusively from how human beings perceive and form relations with other entities. As we have seen, moral standing is here derived from how something is treated, and not what it is. This means that humans are key to determining value, as it is how entities are treated and perceived by humans that determine their moral standing (Gunkel, 2018b). While this surely opens the door for moral standing for robots that are able to mobilize human social instincts and trigger social responses (Sætra, 2020), it is hard to see how this constitutes a form of non-anthropocentrism. On the contrary, it seems like a clear representation of a system based on the axiological anthropocentrism defined in *Axiological Anthropocentrism*. It is interesting to note that I here levy the same kind of criticism against relationalism that Gunkel (2018a, p. 95) uses as an objection against Darling. (2016a): “because what ultimately matters is how “we” see things, this proposal remains thoroughly anthropocentric and instrumentalizes others.” While Gunkel imagines the other as something more than a mirror-image, using Levina’s theory to modify our understanding of the other arguably does not introduce reciprocity or a true recognition of the other for their own sake, since it is human perceptions and experience of the other that is used as the basis for determining value. Thus, relationalism is subject to the very same critique aimed by its proponent on another theory.

Two different anthropocentric doctrines could be developed from relationalism. One is similar to the relationism of Palmer. (2010), in which relations with actual entities are constitutive of moral responsibilities. In the case of robots, I might interact with a Paro robotic seal (Paro Robots, 2021), for example, and feel that I have developed a certain rapport with this entity. This would in turn create responsibilities towards that particular robot, but not toward other Paros. The other approach would be to argue on a more detached level that actual relations are not relevant, while the potential to form relations is Such a doctrine would entail that if humans are capable of forming relationships with Paro robots, then all Paro robots must be awarded moral consideration. This, however, would take us right back to the properties-based approach that the relationists purportedly want to move past.

Of central importance, however, is the fact that the relations being described by the robot ethicists are arguably not really based on true relations at all, as the emphasis is not on mutuality but on how humans perceive and treat other entities. What occurs in the other entity is seemingly irrelevant, and further highlights the relatively extreme anthropocentric nature of the theory. It must be noted that this stands in contrast to care ethics and relational ethics as established in the domain of animal ethics, in which mutuality is a fundamental part of any relationship worth considering. In robot relationism, mutuality and considerations regarding the capabilities, intentions, experiences, etc., of the other is excluded from the analysis, and this leaves us with a peculiar one-sided approach to relations that gives rise to my challenges.

Also of importance is the fact that anthropocentrism is not necessarily a bad thing, once properly understood. Non-anthropocentrism is a term mired in difficulty, as some argue that it is impossible for humans to avoid being anthropocentric, as the very notion of value–either instrumental or intrinsic–is necessarily based on a human perspective (Hayward, 1997; Hargrove, 2003). If robot rights theorists accept this view, however, they might be better off by using Hargrove’s term weak anthropocentrism (2003) to describe their own theory, and argue why this is preferable to strong anthropocentrism. This would dramatically clarify these debates and would be an improvement over a situation in which anthropocentrism alone is assumed to provide sufficient clarity. It does not, and consequently needs to be further elaborated.

#### Properties Strike Back

My second objection is that relationalism is in reality a camouflaged variety of the properties-based approach. This is so because how we relate to other entities is determined by the properties of these others. At the very least, as Gellers. (2020) acknowledges, it significantly influences relations. However, we cannot arguably perceive someone’s true nature, intentions, feelings, etc., so how are the perceived relations with others arrived at?

As discussed in the previous challenge, proponents of relationism generally tend to argue that we need not consider the “internal” properties of robots. If a robot acts in ways that allow it to engage in the kinds of social interactions with humans that the relationists deem important, this is sufficient (Tavani, 2018). This, again, relates to what Danaher (2020) calls ethical behaviorism, which entails that moral duties and responsibilities are grounded in external and observable action, and not entitie’s internal processes and mechanisms.

However, how we relate to someone, and how an entity acts, is dependent on their properties. I might, for example, say that I do not care what species something is, but will evaluate moral standing merely by how I relate to it. The problem, then, is that this will often entail providing moral standing to exactly the same entities as before because those with the properties of humans are the ones I relate to in the manner I consider to be constitutive of moral standing.

It is easy to see why relationalism has emerged so clearly in the discourse on robot rights, as robots are now designed with a range of exactly those properties that are conducive to social relations (Sætra, 2020). It is also an approach that takes us past what might be labeled biological chauvinism, as traditional theories, both anthropocentric and non-anthropocentric, have often focused on the biological foundation of life and moral standing (Gellers, 2020; Manzotti and Jeschke, 2016). Emphasizing biology is indeed problematic, as it excludes mechanical robots from consideration, while it also introduces problems related to the status of humans who integrate with non-biological technology (Sætra, 2019).

Once again, this creates the foundation for two different strands of relationalism. One in which actual properties and capacities required for mutual and reciprocated relationships are used as the basis of determining the potential for relationships, and one in which perceived properties are taken into consideration. The latter strand gives rise to the third objection described below, while the former arguably excludes even the most sophisticated biomimetic robots as parties to relationships.

Relationalism is at times also argued to be able to account for changing social relations and social constructs (Gunkel, 2018b), and this is perceived as an advantage of the approach. In response, proponents of properties-based theories could point out that this is also the case for traditional properties-based approaches, as phenomena like rationality, sentience, consciousness, etc., are also social constructs that change over time, with clear consequences for who and what are accorded moral worth.

#### Edges Cases and the Problem of Anthropomorphism

My third challenge is that any approach to moral standing based on one-sided “relations” based exclusively on perceived properties, coupled with the human tendency to anthropomorphize other entities, leads to potentially absurd implications when the theory is applied in practice. Anthropomorphism describes the process of attributing human properties to other things, i.e. robots, and this can occur intentionally or unintentionally (Coeckelbergh, 2021). The situation that ensues is one in which humans might anthropomorphize other entities, and consequently feel that they relate to these things, which in turn triggers the relationist inclination to use this to accord the thing moral standing.

People anthropomorphize social robots and tend to attribute various traits such as purpose, intentions, etc. to them, despite these robots not actually having such traits of capabilities (Sætra, 2021a). But people also anthropomorphize a wide range of less sophisticated things. A volleyball, for example, in the movie Cast Away, but also a wide range of other things, such as computers, dolls, etc. (Reeves and Nass, 1996; Levy, 2009; Darling, 2016b). In such situations, who is to decide whether these relations, which people according to their own subjective experience are forming with these things, are constitutive of moral standing for the non-human part of the relationship or not?

Are we to rely on objective evaluations of which relationships should matter? If so, the process and criteria by which to perform such evaluations–and who will perform them–become important questions that provide the ground for much potential conflict. On the other hand, if subjective evaluations are to be given consideration, we might in fact end up with a form of subjective relationism where robots are provided moral standing. But so is potentially a volleyball, and a child’s security blanket. As a basis for arriving at a universal theory for determining moral standing, an approach with arguably absurd implications seem to require some more development before it is workable. At the very least, it highlights how an ethical theory of moral standing such as relationalism does not give rise to objective and universal outcomes, but rather show the political nature of deciding who in the end decides.

## Conclusion

The question I have sought to answer is whether relationalism can help move us past chauvinistic and anthropocentric moral theories. I have accepted that relationalism can indeed be effective in allowing non-humans to be awarded moral standing, but I have also argued that the method by which it does so is beleaguered by certain fundamental problems. Firstly, that it is anthropocentric, much like the theories it seeks to replace. Secondly, that it is based on traits, either objectively or subjectively assessed. Thirdly, that anthropomorphism potentially leads to absurdities whenever relationism is used as the basis of determining moral standing without combining relationism with a properties-based approach.

Relationalism is, I argue, based on *Axiological Anthropocentrism*. However, as the theory is not premised on the explicit centering of human perception of value as the basis of moral standing, and since it is also proclaimed to be a solution to the problems of both traditional anthropocentric and non-anthropocentric theories, the theory will be labeled neo-anthropocentric–a type of new anthropocentrism. The novelty of the theory is that it dismisses all explicit references to the superior moral status of humans or human instrumental value as the basis of moral valuation, which means that it is not based on ethical anthropocentrism and human chauvinism. However, it also rejects non-anthropocentric theorie’s adherence to concepts such as inherent value or biocentric egalitarianism. Relationism, then, might not take us past anthropocentrism, but it does take us past human chauvinism. I have also suggested that its proponents might highlight the superiority of weak over strong anthropocentrism (Hargrove, 2003), and Hayward. (1997) has similarly argued that it is not anthropocentrism itself which is the problem, but the various forms of speciesism and human chauvinism.

Relationist neo-anthropocentrism allows us to explore the potential of social ecology as the basis for determining moral standing, and this is indeed valuable, as also shown through traditional approaches to relational ethics and care ethics. However, in contrast to relational non-anthropocentric approaches, such as deep ecology, this form of social ecology explicitly centers humans as their treatment of and relations with others give rise to the other’s moral standing. Relationalism as it is used in the robot ethics discourse provides an interesting theoretical path towards providing robots with moral standing. It is, however, beleaguered by a number of challenges, and this article is intended as a challenge and a request for further elaboration of the approach and theories based on this approach in order to clarify and more clearly position the theory in relation to other related theories and concepts from environmental ethics, such as anthropocentrism. This is not to say that proponents of a relational ethics have not acknowledged the challenges and complexities associated with relationalism–they have–but merely to state that there are still questions that need answering and clarifications that must be made.

On a closing note, some proponents of relationalism might come to accept the label of neo-anthropocentrism and their reliance on a traits-based approach. However, a consequence of this would be that some of the purported advantages of relationism–such as removing us from human chauvinism and the problematic focus on traits–would have to be abandoned. Gellers (2020, p. 153) has to some extent done just this, as he argues that his “explicitly relational” approach must to some extent be combined with the properties-based approach, even if this reintroduces some of the problems associated with properties as a basis of moral standing. And, while it may not be chauvinistic, it is anthropocentric.

## Data Availability

The original contributions presented in the study are included in the article/supplementary material, further inquiries can be directed to the corresponding author.

## Footnotes

1 One distinction I will not pursue in this article is that between short- and long-term anthropocentrism, which distinguishes between those that believe only humans that live right now have moral standing and those that consider potential future humans as well

2 In the following, I will mainly refer to these theories as relationalism, unless specifically pointing to the various names the different authors themselves use. It is also worth noting that it seems unfortunate to speak of these theories as “relational ethic," as that is an already-existing field of study focusing on ethical conduct in various relationships, such as nurse-patient relationships (Ellis, 2007).
